# Causes and contexts of childhood overweight and obesity in the Pacific region: a scoping review

**DOI:** 10.12688/openreseurope.15361.1

**Published:** 2023-04-03

**Authors:** Solene Bertrand-Protat, Juliana Chen, Aurélie Jonquoy, Stéphane Frayon, Si Thu Win Tin, Amerita Ravuvu, Corinne Caillaud, Olivier Galy

**Affiliations:** 1 Interdisciplinary Laboratory of Research in Education, EA 7483, University of New Caledonia, Noumea, New Caledonia; 2 Pacific Community, 95 Promenade Roger Laroque, Noumea, New Caledonia; 3 Charles Perkins Centre, The University of Sydney, Sydney, NSW, Australia; 4 Discipline of Nutrition and Dietetics, Susan Wakil School of Nursing and Midwifery, Faculty of Medicine and Health, The University of Sydney, Sydney, NSW, Australia; 5 Discipline of Biomedical Informatics and Digital Health, School of Medical Sciences, Faculty of Medicine and Health, The University of Sydney, Sydney, NSW, Australia

**Keywords:** Children, Body Mass Index, lifestyle, physical activity, diet, sleep, surveillance, non-communicable diseases, root causes, Melanesia, Polynesia, Micronesia.

## Abstract

**Background:** Non-communicable diseases (NCDs) are a major threat to health and development and account for 75% of deaths in the Pacific Islands Countries and Territories (PICTs). Childhood obesity has been identified as a main risk factor for NCDs later in life. This review compiled overweight and obesity (OWOB) prevalence (anthropometric data) for children aged six to 12 years old living in the Pacific region and identified possible related causes.

**Methods:** We conducted a systematic search using PubMed, Google Scholar and ScienceDirect for articles published between January 1980 and August 2022. We also searched for technical reports from Ministries of Health. Guided by the eligibility criteria, two authors independently read the selected articles and reports to extract and summarise relevant information related to overweight and obesity.

**Results:** We selected 25 articles, two worldwide analyses of population-based studies and four national reports. Information revealed that childhood OWOB prevalence reached 55% in some PICTs. This review also indicated that age, gender and ethnicity were linked to children’s weight status, while dietary practices, sleep time and level of physical activity played a role in OWOB development, as well as the living environment (socio-economic status and food availability), parenting practices and education level.

**Conclusion:** This review highlighted that anthropometric data are limited and that comparisons are difficult due to the paucity of surveys and non-standardized methodology. Main causes of overweight and obesity are attributed to individual characteristics of children and behavioural patterns, children’s socio-economic environment, parenting practices and educational level. Reinforcement of surveillance with standardised tools and metrics adapted to the Pacific region is crucial and further research is warranted to better understand root causes of childhood OWOB in the Pacific islands. More robust and standardized anthropometric data would enable improvements in national strategies, multisectoral responses and innovative interventions to prevent and control NCDs.

## Plain language summary

In the Pacific region, populations have gained faster access to modern lifestyles in the past few decades, causing fundamental changes in the way people move about and eat (including food choices, physical activity, and sedentary time) and a dramatic increase in noncommunicable diseases. This is mainly the case in young generations since they are particularly exposed to an environment that can drive to overweight and obesity. This scoping review aims to understand the causes of overweight and obesity prevalence for children aged six to 12 years old living in the Pacific region and identified possible related causes. This work highlighted that causes of overweight and obesity are mainly attributed to individual characteristics of children and behavioural patterns, children’s socio-economic environment, parenting practices and educational level.

## Introduction

The burden of Non-Communicable Diseases (NCDs) is growing swiftly and is a major threat to health, social and economic development, particularly in low and middle-income countries where resources are often limited ^1^. NCDs can cause severe disabilities impacting individuals’ quality of life and leading to premature deaths. They also present a heavy burden to health care systems and challenge the achievement of the Sustainable Development Goals ^1^. NCDs are generally associated with adulthood, but can develop during childhood and adolescence ^2^.

Childhood obesity in particular, is reaching alarming proportions in many countries and is a strong predictor of adult obesity, which ultimately leads to NCDs such as type two diabetes and cardiovascular diseases ^3^. The World Health Organization (WHO), estimates that 332 million children aged 5–19 years live with overweight or obesity worldwide in 2016 ^4^. Overweight and obesity are defined as abnormal or excessive fat accumulation that may impair health. Body mass index (BMI) and specific growth charts are commonly used to determine childhood weight status. Worldwide comparison of anthropometric data showed that the highest mean body mass index (BMI) in children aged five–nine and 10–19 years old was observed in the Pacific region, with an obesity prevalence of over 30% in some countries ^5^.

**Figure 1.**
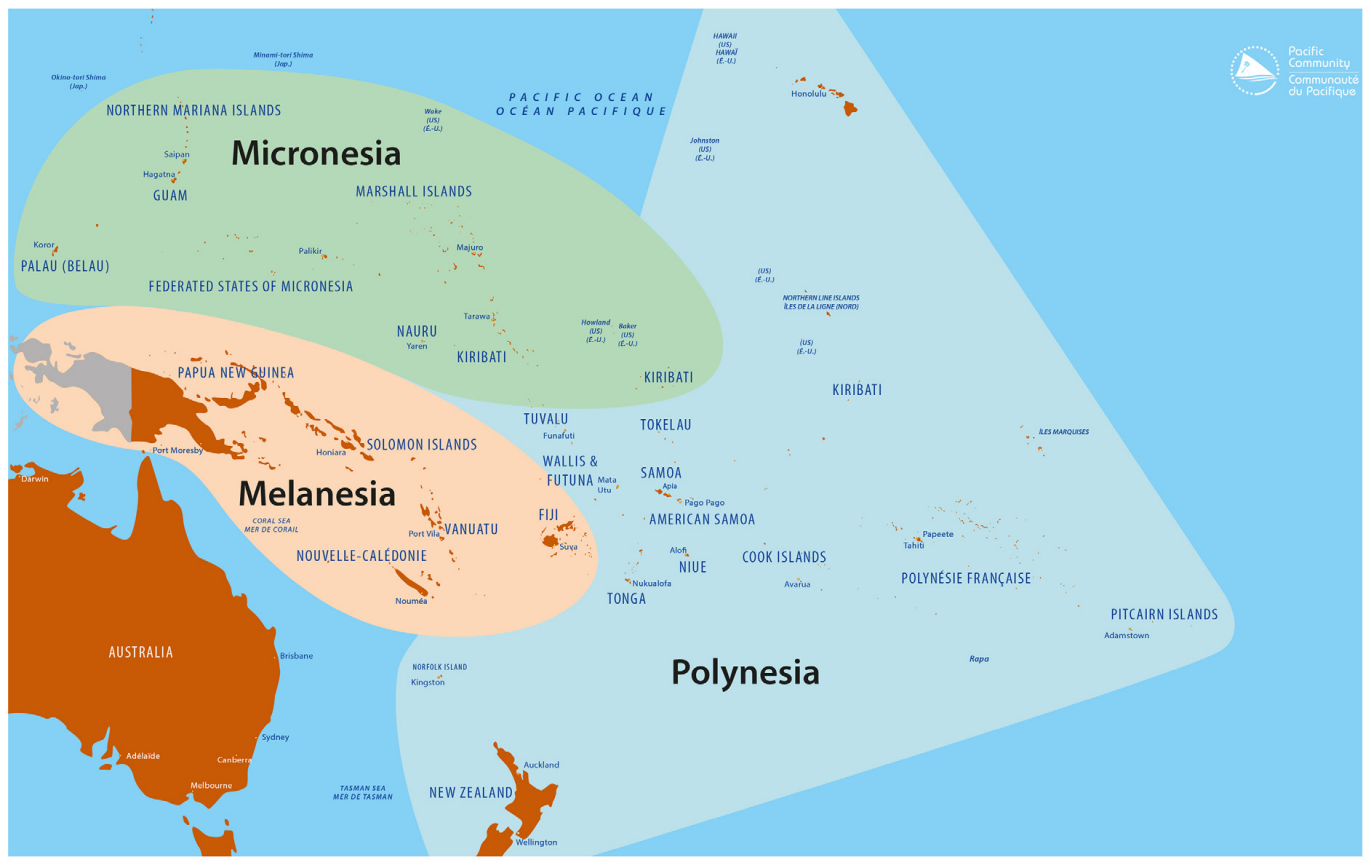
Present Pacific region including Micronesia, Polynesia and Melanesia. *Source: Prepared by the Publishing Team, Pacific Community (SPC), 2022*.

The Pacific region includes 22 Pacific Island Countries and Territories (PICTs) generally grouped into three geographical and cultural zones: Micronesia, includes the Commonwealth of the Northern Mariana Islands, Palau, the Federated States of Micronesia, Kiribati, the Republic of Marshall Islands, Guam and Nauru; Melanesia covers the region encompassing Papua New Guinea, the Solomon Islands, Vanuatu, Fiji, and New Caledonia; and Polynesia which includes Tuvalu, Tokelau, Wallis and Futuna, Tonga, Samoa, American Samoa, Niue, the Cook Islands, French Polynesia and Pitcairn (see Figure 1). Many are sovereign states, but some are associated states or territories of other nations ^1^ ^6^. Due to immigration flows, the inhabitants of PICTs, referred to hereafter as “Pacific islanders”, have formed important diasporic communities in developed Pacific rim countries, such as New Zealand, Australia and USA, especially the state of Hawai’i ^7^.

The prevalence of obesity is higher in Pacific islanders compared to other ethnic groups living in the Pacific region ^8^. Within the Pacific islanders, anthropometric data showed that Polynesians have higher BMI than Melanesians, in both adults and adolescents ^9, 10^. Furthermore, the results of the 2002 National Children’s Nutrition survey conducted in New Zealand children aged five to 14 years old revealed that extreme obesity affects one in 10 Pacific islander children, compared to one in 100 children from New Zealand with European origin ^11^.

With more than 5,500,000 inhabitants under 19 years old in the Pacific region ^12^, the issue of overweight and obesity during childhood requires urgent public health attention. Regional organizations are supporting PICTs in implementing standardized surveys to monitor the health of children in the Pacific region. These include anthropometric data for adolescents (13–18 years old) through the WHO Global School Based Health Survey, information about the BMI of children under five years old and adolescents/adults 15 years and over from Demographic Health Surveys supported by the Asian Development Bank and the Pacific Community, overweight/obesity and diabetes in the 15–17 year age group through the WHO supported STEPwise approach to surveillance surveys (STEPS) and BMI data in children aged 13–17 years in the Health Behaviour and Lifestyle of Pacific Youth Surveys (HBLPY). These surveys all capture data for the five year olds and under and 13–17 years age categories. However, there is lack of reported data for children of primary school age (six to 12 years old). Collecting anthropometric data for this age group is therefore a priority to improve the prevention of childhood obesity ^13^.

To address childhood overweight and obesity, it is important to monitor children’s BMI to assess trends and drive interventions and policies, but it is also critical to identify the root causes. Unhealthy eating habits and an insufficient level of physical activity are often mentioned in relation to childhood obesity ^14^, as well as low sleep duration and high screen time use ^15^. However, the behaviour of children is not enough to explain the development of obesity. Childhood obesity is also linked to social, economic, and environmental determinants including family behaviours, education, food availability, transport, accessibility to sports facilities, food and beverages marketing strategies ^16, 17^. In some US-Affiliated Pacific Islands (USAPI), the Youth Risk Behavior Surveillance System (YRBSS) has been used to monitors six categories of health-related behaviours among adolescents aged 13–18 years old. The Children’s Healthy Living Program for remote and underserved minority populations in the Pacific (CHL) has been monitoring the prevalence of overweight and obesity in children aged 2 to 8 years. The latter has also been monitoring the implementation of interventions addressing policy, environment, messaging, training, and those interventions targeting behaviours including sleep time, screen time, physical activity, intake of fruits and vegetables, water and sugar-sweetened beverages ^18^. Despite these, to date, there are a limited number of studies evaluating the causes of childhood overweight and obesity of children in PICTs.

Therefore, the aim of this review was to conduct a comprehensive review of all available information regarding overweight and obesity prevalence in PICTs for children aged six to 12 years old and to compile current understanding of the causes of childhood overweight and obesity in the Pacific region.

## Methods

We conducted a systematic search of peer-reviewed articles published between January 1980 and August 2022. PubMed, Google Scholar and ScienceDirect databases were searched using the keywords “overweight or obesity,” “children,” and “Pacific islands”. We also conducted a search with specific terms (anthropometry, nutritional status, childhood obesity determinants, childhood obesity root causes, childhood obesity risk factors), individual PICTs names (American Samoa, Cook Islands, Fiji, French Polynesia, Guam, Kiribati, New Caledonia, Niue, Commonwealth of the Northern Mariana Islands, Palau, Papua New Guinea, Pitcairn Islands, Samoa, Solomon Islands, Tokelau, Tonga, Marshall Islands, Federated States of Micronesia, Nauru, Tuvalu, Vanuatu, and Wallis and Futuna) and subregions in the Pacific (Melanesia, Micronesia and Polynesia). A similar search was conducted in French as it is an official language in four PICTs (French Polynesia, Wallis and Futuna, New Caledonia and Vanuatu). Articles were all screened by title and abstract according to the inclusion criteria (see Table 1). Relevant full-text articles were retrieved and included in the review. The search also included technical reports from authoritative sources *e.g.* the Health Ministry of PICTs. Two authors independently read all the selected articles and reports to extract and summarise relevant anthropometric data (see Table 2). If any uncertainty for inclusion, a discussion was made and resolved with a third author.

**Table 1.** Inclusion criteria for the selection of articles.

1. The study was conducted in at least one of the 22 Pacific Islands Countries and Territories (PICTs) and/or New Zealand or Hawai’i
2. Children aged six to 12 years old were included in the study
3. Overweight or obesity was a primary outcome variable and/or at least one determinant or correlate of overweight or obesity was identified
4. Articles from New Zealand and Hawai’i included Pacific islanders and explored determinants or correlates of overweight or obesity.

**Table 2.** Summary of anthropometric data extracted from identified relevant articles (n=27) and national reports available (n=4) for Pacific Islands Countries and Territories (PICTs).

		Author (year)	Setting	Study design (study year)	Population	BMI reference used	Results (Percentages report prevalence)	Overweight or obesity determinants explored
**Worldwide**	**1**	**The GBD** ** 2013 Obesity** ** Collaboration** ** (2015)** ^19^	Worldwide (188 countries)	Pool of 1769 surveys, reports and published studies (1980 to 2012) reporting on prevalence of overweight and obesity based on BMI	2 to 80 years old	IOTF	Globally, the prevalence of overweight and obesity combined has risen by 47.1% for children between 1980 and 2013. In Oceania: Overweight: 20.35% Obesity: 5.35%	_
	**2**	**NCD Risk** ** Factor ** **Collaboration** ** (2017)** ^5^	Worldwide (200 countries and territories)	Pool of 2416 population-based studies (1975 to 2016) with measurements of height and weight	128.9 million participants aged 5 years and older, including 31.5 million aged 5–19 years.	WHO	Prevalence of obesity was more than 30% in girls (5–19 years old) in Nauru, Cook Islands and Palau, and boys in Cook Islands, Nauru, Palau, Niue, American Samoa in 2016. Polynesians and Micronesians had the highest mean BMI in those aged 5–9 and 10–19 years.	_
**Melanesia**	**3**	**Moase *et al.*** ** (1988)** ^20^	PNG (Goodenough Island)	Cross-sectional (1982–1983)	1028 participants (primary school)	WHO	15% of the student population were above standard weight/height	_
	**4**	**Dancause ** ***et al.* (2011)** ^21^	Vanuatu: 3 islands (Ambae, Efate, Aneityum)	Cross-sectional (2007)	375 children aged 6–12 years	WHO	Overweight or obesity in boys: 4.5% Overweight or obesity in girls: 6.3%.	
	**5**	**Weitz *et al.*** ** (2012)** ^22^	8 islands from PNG and Solomon Islands	Cross-sectional and longitudinal (1966–1986)	2000 participants From birth to 35 years old	CDC	BMI cross-sectional comparisons for 3 time periods: 1966–1970 / 1978–1980 / 1985 reveals that the prevalence of overweight and obesity increased substantially during the period of this study among young adults, particularly women, and in groups with more Polynesian affinities, where the frequency of overweight tripled over this 20-year interval. However, the BMI of the more Papuan groups on Bougainville remained remarkably stable.	_
	**6**	**Tubert-** **Jeannin *et al.*** ** (2018)** ^23^	New Caledonia	Cross-sectional (2011–2012)	3138 children aged 6–12 years old	WHO, IOTF	At 6 years: Overweight: 10.8%, obesity: 7.8% (WHO) At 9 years: Overweight: 18.1%, obesity: 11.4% (WHO). At 12 years Overweight: 22.2%, obesity: 20.5% (WHO) Overweight: 25.5 %, obesity: 25.5 (IOTF)	Ethnic group (Polynesian children are particularly at risk for obesity)
	**7**	**Fiji Ministry** ** of Health** ^24^	Fiji	Fiji National Nutrition Survey (2014–2015)	1253 children aged 5–14 years old	WHO	Overweight: 7.2% Obesity: 1.7%	
**Polynesia**	**8**	**Fukuyamo** ***et al.* (2005)** ^25^	Tonga: 2 islands (Tongatapu and Niuamiddleutapu)	Cross-sectional (2002–2003)	895 students aged 5–19 years old	IOTF, CDC	The obesity prevalence for 5–11 years old children living in Tongatapu & Niuatoputapu was, respectively, 7.1% and 7.0% in girls and 2.6% and 2.1% in boys (IOTF). The obesity prevalence for 5–11 years old children living in Tongatapu & Niuatoputapu was, respectively, 10.1% and 10.5% in girls and 5.1% and 6.3% in boys (CDC)	_
	**9**	**Kemmer *et al.*** ** (2008)** ^26^	American Samoa	Cross-sectional (2003)	208 children aged 5–10 years old	CDC	Mean BMI-for-age Z-score: 1.01	-
	**10**	**Bindon *et al.*** ** (1986)** ^27^	Samoa, American Samoa, Hawaii	Cross-sectional (1979–1982)	786 children aged 5.5–11.5 years old	HANES	The children from Western Samoa (traditional) were significantly shorter, lighter and lighter for height than their counterparts in in American Samoa (modern) and Hawaii (migrant).	Modernization, migration
	**11**	**Daigre *et al.*** ** (2012)** ^28^	4 French Overseas Territories: Guadeloupe, Martinique, French Guiana and French Polynesia	Cross-sectional (2007–2008)	101 children from French Polynesia aged 5 – 14 years old included in the study	WHO, IOTF, French references	Overweight: 22.8%, obesity: 20.3% (WHO) Overweight: 17.3%, obesity: 15.9% (IOTF). Overweight and obesity: 31.5% (French references)	_
	**12**	**Ichiho *et al.*** ** (2013)** ^29^	American Samoa	Cross-sectional (2008/2009)	3478 students from kindergarten to grade 11	CDC	Overweight or obesity: 41.3% (grade 2), 43.9% (grade 3) and 50% (grade 5)	
	**13**	**Stewart *et al.*** ** (2014)** ^30^	Cook Islands, New Zealand	Cross-sectional (2012)	267 children aged 1 to 14 years old from Cook Islands	WHO	Mean BMI-SDS: 1	Environmental influences (urbanization)
	**14**	**Veatupu *et al.*** **(2019)** ^31^	Tonga: 1 island (Ha'apai)	Cross-sectional (2017)	35 children aged 10–12 years old	IOTF	Overweight: 14.3% Obesity: 2.9%	_
	**15**	**Thompson** ***et al.* (2019)** ^32^	Samoa	Cross-sectional (2017)	83 children aged 3–7 years old	IOTF, WHO	Overweight: 17% (22.5% for boy; 11.6% for girls), obesity: 4.85% (5% for boys; 4.7% for girls) (IOTF) Overweight: 21.9% (27.5% for boy; 16.3% for girls), obesity: 11.0% (17.5% for boys; 4.6% for girls) (WHO)	Sex differences in the association among nutritional intake and body composition, physical activity was associated with body composition (less %BF),
	**16**	**French** ** Polynesia** ** Ministry of** ** health** ^33^	French Polynesia	Cross-sectional (2014)	1768 students aged 7 to 9 years old	IOTF, French references	Overweight: 35.5%, obesity: 16% (IOTF) Overweight and obesity: 34% (French references)	Skipping breakfast, having snacks during the morning bought from shops/food trucks, to not be registered in a sports club, sleeping less than 10 hours per night.
	**17**	**Department** ** of health and** ** Department ** **of Education ** **of American ** **Samoa** ^34^	American Samoa	Cross-sectional (2008–2009)	3478 students aged 7 to 16 years old	CDC	Overweight or obesity: 55.6%	Inadequate sleep, reliance on vehicles rather than walking to school, and social norms that are skewed toward accepting obesity may be major contributing factors toward the high prevalence of obesity.
	**18**	**Wallis and** ** Futuna health ** **Department** ^35^	Wallis and Futuna	Cross-sectional (2020)	406 students aged 7 to 10 years old	IOTF, WHO, CDC, French references	Overweight: 24.4%, obesity: 26.3% (IOTF) Overweight: 21.4%, obesity: 35.5% (WHO) Overweight: 13.5%, obesity: 43.1% (CDC) Overweight and obesity: 49% (French references)	
**Micronesia**	**19**	**Bruss *et al.*** ** (2005)** ^36^	Commonwealth of the Northern Mariana Islands (Saipan)	Qualitative (2002)	32 participants in focus groups (mothers, fathers, and grandparents of children 6 to 10 years old)		Qualitative data on the perception of childhood obesity within 1 multiethnic community	Influence of sociocultural, familial, and nutritional factors on health care behaviors.
	**20**	**Novotny *et al.*** ** (2007)** ^37^	Commonwealth of the Northern Mariana Islands	Cross-sectional (2005)	420 children aged 6 months – 10 years	CDC	Overweight: 19%	Breastfeeding (children breastfed has lower BMI)
	**21**	**Durand** ** (2007)** ^38^	FSM (Yap)	Cross-sectional (2006)	1736 children aged 2 to 15 years old	WHO	5 to 10 years Overweight: 15% (12% for boys and 19% for girls) Obesity: 19% (both for boys and girls),	
	**22**	**Paulino *et al.*** ** (2008)** ^39^	Commonwealth of the Northern Mariana Islands (Rota, Saipan and Tinian)	Cross-sectional (2005)	393 children aged 6 months to 10 years old	CDC	Overweight or obesity: 26% (4–6 years) and 45% (7–10 years)	
	**23**	**Ichicho *et al.*** ** (2013)** ^40^	Federated States of Micronesia (State of Yap)	Cross-sectional (2008–2009)	Wa'ab community health center household survey (2006–2007): 1736 children Outer island household survey (2008–2009): 2042 children aged 2–14 years Maternal & child health, school health survey (2006–2007): 1245 students from 14 elementary schools Maternal & child health, school health survey in (2009–2010): 1415 students from elementary schools and early childhood education centers	IOTF	Overweight or obesity: 20.5% to 33.8%	
	**24**	**Paulino *et al.*** **(2015)** ^41^	Guam	Cross-sectional (2010–2014)	106 827 students aged 4–19 years old	CDC	Overweight: 16.0% (2010–2011) and 16.5% (2013–2014) Obesity: 23.6% (2010–2011) and 22.6% (2013–2014).	
	**25**	**Paulino *et al.*** ** (2017)** ^42^	FSM, RMI, Palau	Cross-sectional (2013–2015)	1200 children aged 2–8 years old	CDC	Overweight or obesity: 12.9%	
	**26**	**Matanane** ***et al.* (2017)** ^43^	Guam	Cross-sectional (2012–2013)	466 children aged 2 – 8 years old	CDC	Overweight: 16% Obesity: 13%	Lower BMI z-scores in participants having a small market close to their residences.
	**27**	**Passmore ** ***et al.* (2019)** ^44^	Republic of Marshall Islands (Majuro islands)	Cross-sectional (2017–2018)	3,271 children aged 4–16 years old	CDC	Overweight: 8.2% Obesity: 5.1% (4–6 years: 3.3%; 7–9 years: 4.4%, 10–12 years: 7.1%),	Obesity prevalence was higher in boys and in children attending private schools.
	**28**	**Lean** ** Guerrero** ***et al.* (2020)** ^45^	Guam	Cross-sectional (2013)	865 children aged 2–8 years old	CDC	Overweight: 13.39% Obesity: 13.15%	Children with overweight or obesity were more likely to have educated caregivers and consume more sugar sweetened beverages
**Multi PICTs**	**29**	**Novotny *et al.*** ** (2015)** ^46^	USAPI: Hawaii, Alaska, Commonwealth of the Northern Mariana Islands, Guam, American Samoa, Palau, Republic of the Marshall Islands (RMI), 4 Federated States of Micronesia (Pohnpei, Yap, Kosrae, Chuuk)	Systematic review		CDC	At 8 years Obesity: 23% Overweight and obesity: 39%.	
	**30**	**Novotny *et al*.** ** (2016)** ^47^	USAPI: Hawaii, Alaska, Commonwealth of the Northern Mariana Islands, Guam, American Samoa, Palau, Republic of the Marshall Islands (RMI), 4 Federated States of Micronesia (Pohnpei, Yap, Kosrae, Chuuk)	Cross sectional (2013)	5463 children aged 2–8 years old	CDC	Overweight: 14.4%. Obesity: 14.0% (16.3% for 6–8 years old)	race/ethnicity, age
	**31**	**Novotny *et al.*** ** (2017)** ^48^	USAPI: Hawaii, Alaska, Commonwealth of the Northern Mariana Islands, Guam, American Samoa, Palau, Republic of the Marshall Islands (RMI), 4 Federated States of Micronesia (Pohnpei, Yap, Kosrae, Chuuk)	Cross-sectional (2012)	5462 children aged 2 – 8 years old	CDC	Obesity: 14%	sex, race, and jurisdiction income level are associated with obesity

Note : GBD = The collaborative groups of the Global Burden of Disease Study (GBD), NCD = Non-communicable diseases, BMI = Body Mass Index, PNG = Papua New Guinea, WHO = World Health Organization, CDC = Centers for Disease Control And Prevention, IOTF = International Obesity Task Force, HANES = National Health and Nutrition Examination Survey, FSM = Federated States of Micronesia, RMI = Republic of Marshall Islands.

Due to the paucity of surveys that explored childhood obesity and overweight causes in PICTs, the search was extended to New Zealand and Hawai’i. Articles were added only where they were including Pacific islanders and exploring determinants of overweight or obesity as results (see Table 3). Due to the diversity and relatively small number of studies on this topic, no attempt was made to evaluate individual study and there were no restrictions on study design.

**Table 3.** Summary of obesity determinants identified in relevant articles for New Zealand and Hawai’i (n=20).

		Author (year)	Study design (study year)	Population	Obesity determinants identified
**Hawai'i**	**1**	**Brown ** ***et al.* (2011)** ^51^	Cross sectional	125 children: 59 in Kindergarten (mean age 5.6 years old) and 66 in third grade (mean age 8,7 years old)	Ethnic disparity in adiposity occurs after the age of 6 years and is confined to males in this study. For older girls, their father's educational attainment was inversely related to adiposity.
	**2**	**Teranishi *et al.*** ** (2011)** ^52^	Cross-sectional (2007)	874 children 10–17 years of age	Poorer overall health status, gender, race and parental education were significantly associated with overweight/ obesity.
	**3**	**Novotny *et al.*** ** (2013)** ^53^	Cross-sectional (2010)	5–8 years old	Samoan, native Hawaiian, Filipino and mixed ethnic ancestries had higher levels of overweight & obesity than white or Asian population. Higher neighborhood education level was associated with lower BMI. Younger maternal age and lower maternal education were associated with child overweight and obesity.
	**4**	**Braden and** ** Nigg (2016)** ^54^	Narrative review (2000–2015)	Children from birth to 18 years old	Early life and contextual factors (infant-feeding mode, geographic location and education)
	**5**	**Brown *et al.*** ** (2018)** ^55^	Cross-sectional	105 children: 49 in kindergarten (mean age 5.5 years old) and 56 in third grade (mean age 8.6 years old)	In the older cohort, high physical activity levels were significantly related to lower BMI, waist circumference and bodyfat percentage. Inactivity was positively correlated with bodyfat percentage.
	**6**	**Mosley *et al.*** ** (2018)** ^56^	Longitudinal (2001–2003)	148 adolescent girls aged 9–14 years old	Results revealed changes in dietary patterns over time and an association between intake and BMI
	**7**	**Banna *et al.*** ** (2018)** ^57^	Cross-sectional (2015)	84 adolescent girls aged 9–13 years old	There were correlations between cognitive restraint, uncontrolled eating, emotional eating and BMI.
**New Zealand**	**8**	**Utter *et al.*** **(2005)** ^58^	Cross-sectional (2002)	3275 children aged 5 to 14 years old	Children and adolescents who watched the most TV were significantly more likely to be higher consumers of foods most commonly advertised on TV: soft drinks and fruit drinks, some sweets and snacks, and some fast food.
	**9**	**Duncan *et al.*** ** (2006)** ^59^	Cross-sectional	1115 children aged 5 to 12 years old	There was a link between daily steps and body fatness in children.
	**10**	**Utter *et al.*** ** (2007)** ^60^	Cross-sectional (2002)	3275 children aged 5 to 14 years old	Skipping breakfast was associated with a higher BMI. Children who missed breakfast were significantly less likely to meet recommendations for fruit and vegetable consumption and more likely to be frequent consumers of unhealthy snack foods.
	**11**	**Goulding *et al.*** ** (2007)** ^11^	Cross-sectional (2002)	3049 children aged 5 to 14 years old	Ethnic differences in prevalence of extreme obesity: extreme obesity affects 1 in 10 Pacific islander children, 1 in 20 Maori children, versus 1 in 100 New Zealand, European and other.
	**12**	**Duncan *et al.*** ** (2007)** ^61^	Cross-sectional	1229 children aged 5 to 11 years old	Three lifestyle risk factors related to fat status identified: low physical activity, skipping breakfast and insufficient sleep during weekdays.
	**13**	**Rush *et al.*** ** (2010)** ^62^	Longitudinal (2000 – 2006)	722 children from birth to 6 years old	Positive correlation between birth weight and weight at six years.
	**14**	**Hodgkin *et al.*** **(2010)** ^63^	Cross-sectional (2002)	3275 children aged 5 to 15 years old	Rural children had a significantly lower BMI, smaller waist circumferences and thinner skinfold measurements than urban children.
	**15**	**Oliver *et al.*** **(2011)** ^64^	Cross-sectional (2006–2007)	102 children aged 6 years old and their mothers	Watching television every day and having a mother with a high waist circumference were associated with increased body fat z-score.
	**16**	**Carter *et al.*** ** (2011)** ^65^	Longitudinal (2001–2009)	244 children from birth to 7 years old	Young children who do not get enough sleep are at increased risk of becoming overweight. Maternal BMI, ethnicity, smoking during pregnancy, and the intake of non-core foods were all positively associated with BMI.
	**17**	**Williams *et al.*** ** (2012)** ^66^	Comparison of 2 cohorts born 29 years apart	974 participants in cohort 1 (born in 1972–1973) and 241 participants in cohort 2 (born in 2001–2002).	Societal factors such as higher maternal BMI and smoking in pregnancy contribute most to the secular increase in BMI.
	**18**	**Oliver *et al.*** ** (2013)** ^67^	Cross-sectional (2006)	393 children aged 6 years old and their mothers (386)	Watching TV every day and having mother with a high waist circumference is associated with a greater waist circumference
	**19**	**Landhuis *et al.*** ** (2014)** ^68^	Longitudinal (1972–2005)	1037 participants (from birth to 32 years old)	Sleep restriction in childhood increases the long-term risk for obesity.
	**20**	**Tseng *et al.*** ** (2015)** ^69^	Longitudinal (2000 – 2011)	1249 children from birth to 11 years old	Changes in maternal acculturation can influence children's growth, suggesting the importance of lifestyle or behavioral factors related to a mother’s cultural orientation.

## Results

**Figure 2.**
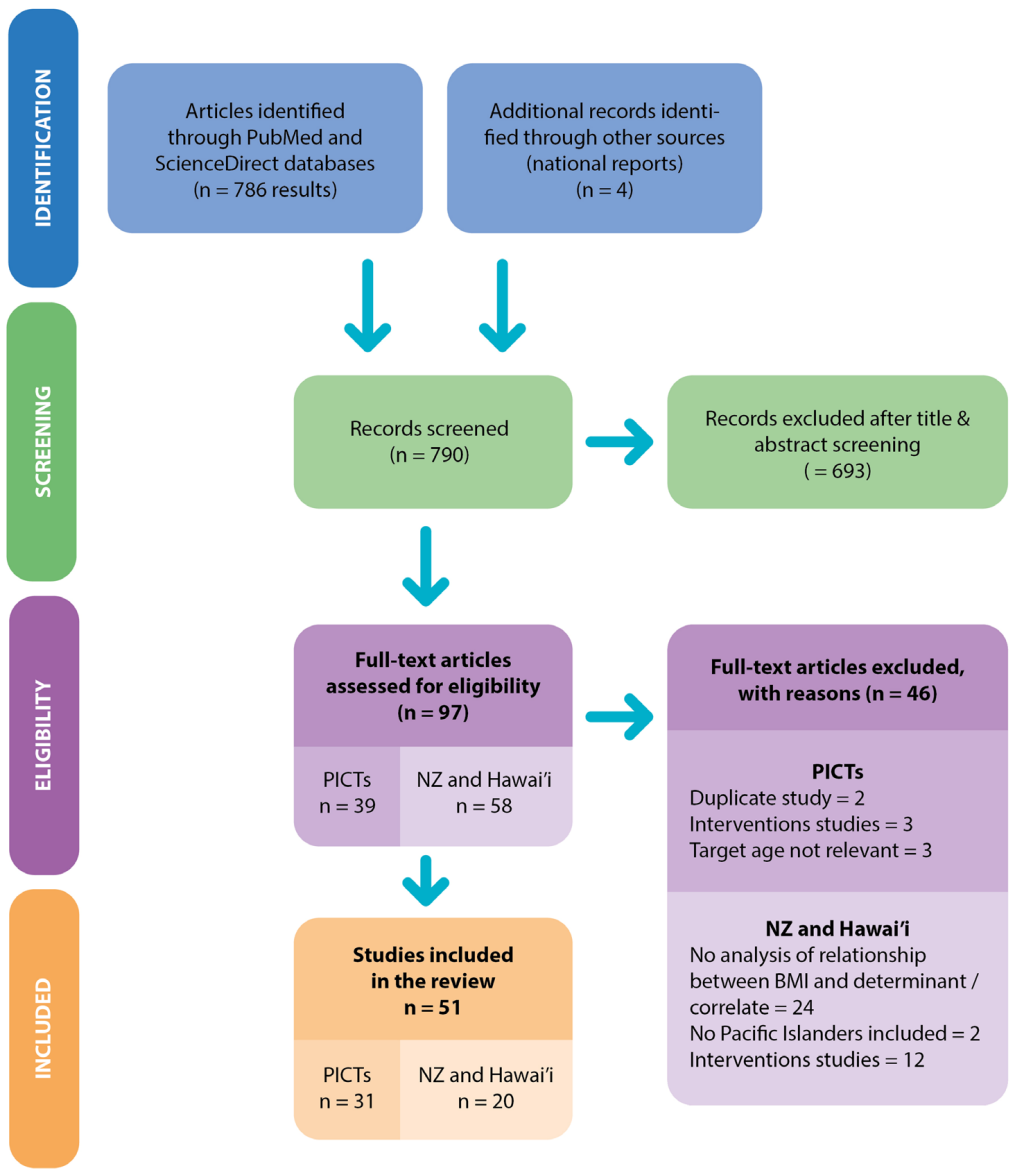
Workflow diagram.

The search retrieved 786 articles and four national reports as shown in the PRISMA-ScR flow diagram in Figure 2 ^49^. The PRISMA-ScR checklist for this study is also publicly available ^50^. After initial screening, 97 documents met the inclusion criteria. There were 35 articles and four national reports reporting studies conducted in PICTs, 14 articles in Hawai’i and 44 in New Zealand. Of these, 46 were excluded because they were related to the same study and provided no additional information, the sample’s age did not meet the criteria ( *e.g.* 2–6 years old or 12–18 years old) or the study reported an intervention.

### Characteristics of included studies from PICTs

Selected articles included: 22 original studies, reported across 25 articles, two worldwide analyses of population-based studies and four national reports (see Table 2).

**Figure 3.**
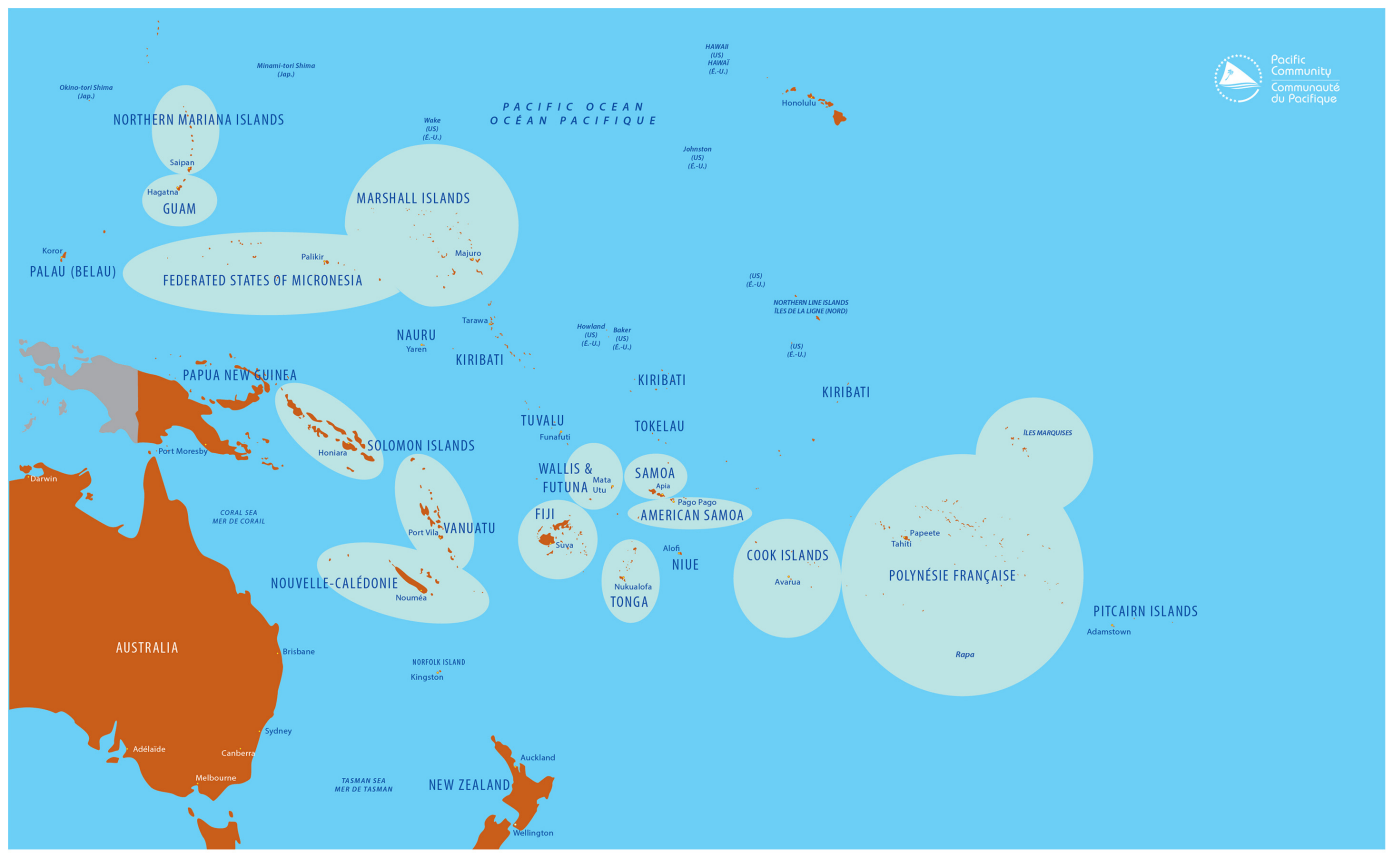
Geographic representation of available anthropometric data on overweight or obesity in Pacific Island countries and territories. Note: For PICTS where BMI data is available, these are marked with a circle on the map. For PICTs where no anthropometric data is available, these are left unmarked. Source: Prepared by the Publications Team, Pacific Community (SPC), 2022.

Of the 31 articles and reports included, 28 were cross-sectional studies, one was an qualitative study, one a systematic review and a blended study (presenting anthropometric data from 3 cross-sectional and 1 longitudinal study). The sample size in these studies ranged from 32 to 106,827 participants, with half of the studies including 1,000 or less participants or were focused on a very specific location ( *e.g.* one island or one village/province). In terms of the study setting, two studies were global studies ^5, 19^, one focused on the USAPI ^47, 48^, five were implemented in Melanesian PICTs ^20– 24^, eleven in Polynesian PICTs ^25– 35^ and ten in Micronesian PICTs ^29, 36– 39, 41– 45^ (see Figure 3). Six studies aimed to monitor childhood obesity at a national level: Guam, New Caledonia, Fiji, French Polynesia, Wallis-and-Futuna and American Samoa ^23, 24, 33– 35, 41^. These national studies were conducted in school settings and included all students, or a proportionate-to-population sized cluster samples. But most of the studies accessed the children through communities/households. In some studies, the main objective was to explore other health conditions (anaemia, oral health, acanthosis nigricans, *etc.*) rather than in measuring overweight/obesity prevalence ^23, 26, 47^.

All included studies reported on measured anthropometric data (no self-report), however, no consistent reference method was used. Across the studies, the prevalence of overweight and obesity was measured using WHO (n=10), Centre for Diseases Control and Prevention (CDC) (n=16), International Obesity Task Force (IOTF) (n=9) or French BMI reference standards (n=3). Due to the number of different child growth references available, studies performed in PICTs often presented anthropometric data using two (or even sometimes four) reference standards to allow for comparison with other studies. The northern territories (American territories or US associated territories) used the CDC reference standards only. There were no articles reporting on anthropometric data from Tokelau, Palau, Tuvalu, Niue, Kiribati, Nauru and Pitcairn (see Figure 3).

### Childhood overweight and obesity prevalence in PICTs reported in the articles

Due to the diversity of the results presented in the articles, we chose to focus on reporting on the outcome of excess weight (overweight and obesity both included) hereafter identified as OWOB. Where possible and relevant, overweight and obesity data are presented separately for more precision (see Table 2).

According to the studies included in this review, overall childhood OWOB prevalence in PICTs reached 40% in Micronesia and was above 55% in Polynesia. For the Melanesian areas, obesity affecting 1.7% of the children in Fiji and up to 25% in New Caledonia. Obesity was ranked between 5.1% and 23.6% in Micronesia and was above 40% in Polynesian countries.

### Childhood overweight and obesity causes identified

Thirteen of the articles identified at least one determinant of childhood overweight and obesity ^23, 27, 30, 32– 34, 36, 37, 43– 45, 47, 48^. One qualitative study focused on childhood obesity determinants ^38^.

**Figure 4.**
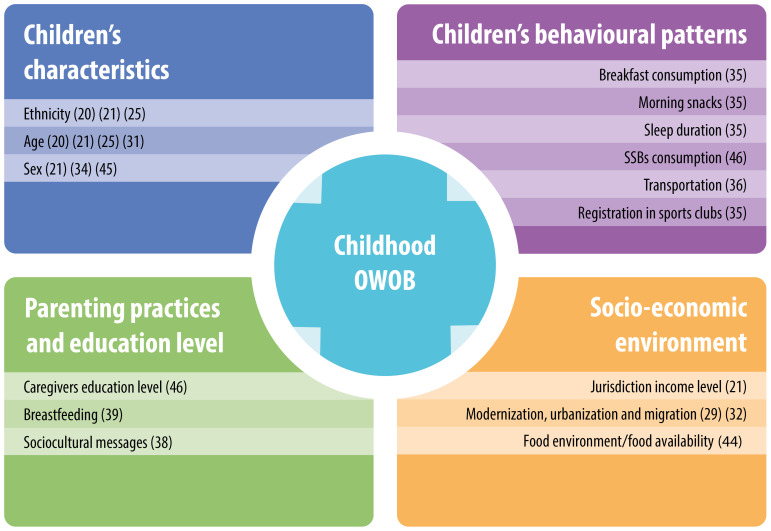
Main causes of childhood overweight and obesity identified in PICTs. Note: References to studies are indicated in brackets.

Determinants identified can be divided into four main subgroups: children’s characteristics, children’s behavioural patterns, parenting practises/education level and socio-economic environment ( Figure 4).

- ***Children’s characteristics:*** Ethnicity was associated with BMI, with Polynesians found to have higher BMIs than other Pacific islanders. Indeed, results from pluri-ethnic PICTs show that Polynesian children are particularly at risk of obesity. For instance, in New Caledonia, obesity rates are 22.1% for Melanesians and 25.1% for Polynesian children ^23^. In the USAPI study ^47, 48^, prevalence of obesity varied among Pacific race/ethnic groups, with Polynesians found to have a higher rate of obesity than Micronesians. A similar trend for ethnicity was also observed in surveys conducted in New Zealand and Hawai’i. Native Hawaiian boys aged 8–9 years old were found to be significantly more overweight than their classmates ^51^. In New Zealand, extreme obesity was found to affect one in 10 Pacific islander children and one in 20 Māori children, compared to one in 100 New Zealand European ^11^.
  Studies with pooled anthropometric data indicated that the prevalence of OWOB increases with age. For instance, obesity rates in New Caledonia were 7.8% at six years old, 11.4% at nine years old and 20.5% at 12 years old ^23^. In the USAPI, children 6–8 years old were more likely to be obese than children 2–5 years old (16.3% compared to 12.9%) ^48^. In American Samoa, the OWOB rate was 41.3% for grade two children, 43.9% for grade three and 50% for grade five ^29^. It was also found that sex might influence weight status. In the USAPI, boys aged 2 to 8 years old were more likely to be obese than girls (16.3% vs 11.6%) ^48^. In Samoa obesity rates were 17.5% for boys and 4.6% for girls based on WHO z-scores for children 3–7 years old ^32^. The results of the National Survey of Children’s Health conducted by the CDC in Hawai’i highlighted that more boys (32.5%) than girls (24.2%) were overweight/obese ^52^.
- ***Children’s behavioural patterns:*** A study conducted in French Polynesia found that the main factors associated with increased risk of OWOB were the absence of breakfast (OR: 1.33 [1.05 –1.69]), having snacks during the morning bought from shops/food trucks (OR: 1.62 [1.25–2.11]), not be registered in a sports club (OR: 1.28 [1.01–1.62]) and less than 10 hours of sleep per night (OR: 1.39 [1.03–1.87]) ^33^. The National Children’s Nutrition Survey implemented in New Zealand indicated that skipping breakfast was associated with a higher BMI in children aged five to 14 years old and that children who missed breakfast were significantly less likely to meet recommendations for fruits and vegetables consumption, and more likely to be frequent consumers of unhealthy snacks ^60^. Similarly, Duncan *et al.* identified three lifestyle risk factors related to fat status in New Zealand children: low physical activity, skipping breakfast and insufficient sleep on weekdays ^70^. The Children’s Healthy Living Study in Guam indicated that compared to healthy weight children, children with OWOB were consuming more sugary sweet beverages (SSBs) ^45^. The survey conducted by the American Samoa Department of Health found that reliance on vehicles rather than walking to school and social norms that were skewed towards accepting obesity are risk factors to OWOB ^34^. This is consistent with results from a study conducted by Brown *et al.* in Hawai’i where high physical activity levels were significantly related to lower BMI, waist circumference and body fat percentage ^55^. In contrast, inactivity was significantly positively correlated with body fat percentage for students grade three (eight years old) ^55^.
- ***Parenting practices and education level:*** Parenting practices and caregivers’ education level were also associated with children weight status. , Novotny *et al.* found that children from Northern Mariana Islands who had been breastfed had significantly lower BMIs than those who were not ^37^. It was also reported that children with overweight or obesity in Guam were more likely to have educated caregivers (over 12th grade) ^45^. However, these findings are inconsistent with the results of studies conducted in Hawai’i, where it was found that the father’s level of educational attainment was inversely related with their daughter’s adiposity ^51^. Similarly, lower maternal education was associated with greater childhood overweight and obesity ^53^, and the National Survey of Children’s Health indicated that the prevalence of OWOB decreased with greater numbers of years of parental education ^52^.
***Socio-economic environment:*** The environment in which children evolve affects their health status. For instance, the food store environment plays a role in the OWOB rate. In Guam, living close to a small market was associated with a lower BMI while children who lived close to a convenience store had a higher BMI ^43^. Children living in a lower to middle income jurisdiction in USAPI were less likely to be obese than those from higher income jurisdiction ^48^. Similarly, in the Marshall Islands, obesity prevalence was higher in children attending private schools ^44^. The results for two comparative studies ^27, 30^ showed that children living in the islands (Samoa and Cook Islands) were less obese than Samoan children living in Hawaii or Cook Islander children living in New Zealand. The studies attributed this to the more traditional ways of life practiced in both Samoa and Cook Islands.

## Discussion

This systematic review provides an overview of the prevalence and determinants of childhood OWOB in PICTs, specifically among 6–12-year-olds. This review found that the prevalence of overweight and obesity reached 55.6% (CDC BMI reference tool) in some PICTs and childhood obesity ranged from 1.7 to 35.5% across the Pacific region (WHO BMI reference tool). The review highlighted that this high prevalence of OWOB in the region was mainly explained by children’s individual characteristics and behavioural patterns, parenting practices and education levels, and children’s socio-economic environment.

### Overweight and obesity prevalence in children living in the pacific region: data availability, tools and methods

The prevalence of OWOB in children aged six to 12 years old observed in this review (see Table 2) is higher than what is observed in high-income countries or related states of the region such as New Zealand where obesity prevalence is 9.4% ( 2–14 years old), 8% in Australia ( 5–14 years old), 18.4% in the United states (6–11 years old) ^71^ and 3.9% in France (6–17 years old) ^72^.

At the same time, this review revealed an incomplete picture of childhood OWOB across the region related to disparity of anthropometric data available, tools and methods used. Among the literature, anthropometric data availability and mechanisms for reporting of child growth monitoring have often been described as key issues that need to be addressed to drive health policies and monitor interventions in PICTs ^13, 73, 74^. In the region over the past decade, more studies have been conducted in Polynesian and English-speaking countries compared to Melanesian and French territories (see Table 2). The presence of research units as well as non-governmental organisations have also likely influenced the way and where anthropometric data are collected. Also, limitations in human and financial resources in PICTs do not always allow national health surveys to be undertaken in a periodic manner to collect valid and reliable anthropometric data that can then inform understanding of the trends of OWOB among these PICTs. Hence, the combination of these factors has contributed to the gap in accessibility and availability of anthropometric data related to childhood OWOB in the Pacific region. Furthermore, we only found a few articles published by local governments and national reports, however these were not readily available/accessible for public use. Collaboration between ministries of health and regional universities should be encouraged to facilitate analysis, publication, and dissemination of results.

Obesity is commonly defined as an excess of fat accumulation that present a risk for health. There are indirect methods available to calculate fat mass such as the dual-energy X-ray absorptiometry (DEXA), bioelectrical impedance analysis (BIA) and densitometry ^75^. However, those methods required specific equipment and/or expertise and are thus inadequate for use in national surveys. Anthropometric measures are less accurate for measuring the excess of body fat, but they are more practical and easier to use to monitor childhood obesity. The most common used ones are subcutaneous skinfolds, height, weight, waist and body circumferences to calculate ratios, percentage of body fat or BMI ^76^. All the studies analysed in this review used BMI to assess the weight status of children. This is likely because it is relatively cheap to collect the anthropometric data and easy to calculate. However, assessing the BMI of children requires consideration of biological maturation. Therefore, children’s BMI is categorised using a variable threshold that considers the child’s age and sex. The most commonly used BMI reference tools are the one provided by WHO ^77^, CDC ^78^ and IOTF ^79^. Each growth reference tends to have a set of recommended thresholds defined by statistical conventions, *e.g.* a whole number of standard deviations from the mean or a whole number of centiles. Studies analysed in this review used a combination of all those references to determine BMI and OWOB in children. Thus, any interpretation of children OWOB at a regional level, remain an estimate if based on existing literature. Therefore, future surveys need to be standardised at a regional level to better monitor childhood obesity in the Pacific region. The COSI Protocol developed in Europe by WHO could be a good starting point ^80^. There is also a need for multi-country studies ( *e.g.* studies that involve at least 2 PICTs) to allow for comparisons between countries. In addition, WHO has acknowledged the need for culturally specific standards because current BMI-for-age charts are not appropriate for Asian and Pacific island children ^81^; implying that any interpretation needs to be cautious and the necessity for this tool to evolve in the future.

### Root causes of overweight and obesity in children living in the Pacific region

This review also highlights some possible root causes of childhood obesity in the Pacific region. For instance, ethnicity plays a major role in the development of obesity, and similar results are found in other countries such as the United States of America where White and Asian American children have significantly lower rates of obesity compared with African American and Hispanic children ^82^. The impact of ethnicity on overweight has also been observed in adolescents in the Pacific region ^83^. This factor should be considered in future research and studies focused on Oceanians of Non-European, Non-Asian Descent (ONENA), which could be relevant in some PICTs ^84^. The prevalence of overweight and obesity has also been found to increase with age, which is consistent with what is observed in other countries such as Australia ^85^. The review also highlights that boys are more affected by obesity than girls in the Pacific for children aged six to 12 years old. Similar findings have been published in other regions, *e.g.* the WHO COSI survey conducted in 36 European countries, where the prevalence of obesity tended to be higher for boys than for girls aged six to nine years old ^80^.

Our findings revealed that lifestyle influences OWOB prevalence in children living in the Pacific region. Studies included in the review showed that diet (especially consumption of SSBs/snacks and, absence of breakfast) was associated with children weight status. The eating habits of Pacific Islanders have been profoundly modified with the establishment of commercial exchanges increasing access to processed products, to the detriment of local healthy foods ^86– 88^. Among dietary habits related to weight gain, the link between consumption of sugary drinks and OWOB has been clearly established ^89, 90^. Studies implemented in the Pacific region have highlighted the high consumption of SSBs by children and adolescents ^45, 91, 92^. WHO recommends SSB taxation as an efficient tool to reduce consumption ^93^. There are SSB taxes in 16 of 21 PICTs ^94^ but more efforts are required especially to ensure that SSBs are not easily accessible to children. Strong school food policies, effective restrictions on food marketing and school or community based interventions are essential ^95– 97^.

Regarding physical activity, information was limited in the articles retrieved for this review. In 2020, WHO released updated recommendations: “children and adolescents should do at least an average of 60 min per day of moderate-to-vigorous physical activity across the week” ^98^. To ensure that children meet those recommendations, governments are strongly encouraged to include physical activity in their national school curriculum. This is currently monitored through the Pacific Monitoring Alliance for NCD Action (MANA) framework. According to the Pacific MANA, 15 PICTs have included physical activity as a compulsory component of the school curriculum ^99^. School interventions that include promotion of daily physical activity also need to be strengthened in the region; like the Healthy Child Promising Future project implemented in Fiji and Wallis-and-Futuna, which assigns 30 minutes of daily physical activity to be included in school time ^100^. Furthermore, the country-driven Pacific Ending Childhood Obesity Network (Pacific ECHO), established in 2017, has a strategic priority area focused on the development of a region-wide physical activity campaign and aims to support physical activity interventions for children ^74^.

As part of healthy lifestyles, this review found that sleep duration was linked to childhood OWOB. The association between sleep and weight status is well documented in the literature ^99^, but there is limited anthropometric data available in the Pacific. WHO has released sleep time guidelines only for children under five years ^100^. However, based on anthropometric data collected in USAPI, the CHL program has developed tools for communities and tips for caregivers to increase children’s sleep time using CDC recommendations. Awareness campaigns and interventions related to sleep duration should be widely organized in the region.

High intakes of calories, lack of physical activity and hours of sleep are leading to increased weight in children population. Future research needs to focus on social cultural factors that influence children’s lifestyle. The Pacific Obesity Prevention in Communities (OPIC) project paved the way by exploring social structures, values, beliefs, perceptions, attitudes and expectations which have a significant influence on Fijian and Tongan adolescents’ individual behaviours related to eating, activity and body image ^101^.

 Breastfeeding appears to be a protective factor regarding OWOB in our review. WHO and UNICEF recommend exclusive breastfeeding for six months to achieve optimal growth, development and health. According to the State of the World’s Children 2016 data, 55% of children are exclusively breastfed during the first six month after birth in the Pacific ^101^, with disparities between countries (74% in Solomon Islands to 31% in Republic of Marshall Islands); but still higher than what is observed in Pacific islands families living in New Zealand ^102^. To maintain good rates of breastfeeding, PICTs should implement measures to regulate the promotion of breast-milk substitutes ^103^.

The role of the environment in children’s weight status needs to be considered in too. The income level of children’s living area is associated with OWOB rates in studies conducted in USAPI. This finding can be extended at regional level by using the World Bank country classifications by income level PICTs with lower income levels were less affected by overweight and obesity. This can be explained by the lack of financial means of households, which encourage family farming activities rather than buying often processed or highly processed food from supermarkets or convenience stores. Affluence leads to the purchase of unhealthy food products and gives access to technologies that promote a sedentary lifestyle. This has been already observed in 104, 105.

In our analysis, we draw special attention to important knowledge deficits on the topic of OWOB and its roots causes in children living in the Pacific region. More research is required to better understand socio-cultural determinants of childhood OWOB. Among the 47 articles reviewed, only one qualitative study was listed. Indeed, qualitative analysis is essential to identify risk factors that might be specific to the region and have not been explored elsewhere and/or observed with quantitative surveys. So, it is relevant to set up mixed longitudinal studies such as the Pacific Islands Families Study ^106^ implemented in New Zealand among children of Pacific Islanders exclusively to study the evolution of the anthropometric characteristics of children during their growth, but also social determinants that could explain overweight and obesity. Our scoping review has some limitations. We made the choice to add grey literature through reports available online. Unfortunately, national reports are usually not accessible for public use. Nonetheless, this review provides an overview of the available anthropometric data on OWOB prevalence for children between six to 12 years old and the current root causes identified in the Pacific region.

## Conclusion

The results of this review indicate that unhealthy behaviours and lifestyles are prevalent in children and brings new information on the causes of obesity in children in an understudied population. The study illustrates concerning trends particularly with the prevalence of overweight and obesity reaching up to 55% in some PICTs and childhood obesity ranging from 1.7% to 35.5% across the Pacific region. These trends are attributed to the individual characteristics of children and behavioural patterns, parenting practices and educational level, and children’s socio-economic environment. Although anthropometric data was limited and comparisons difficult due to the paucity of surveys and the varying range of tools and methods used to monitor childhood OWOB, this review highlights the critical need for more robust anthropometric data and more qualitative studies to explore childhood OWOB root causes. This will provide a more nuanced understanding of the environments and communities children operate in and provides opportunities to interrogate further how their choices are shaped. This will better inform the development of suitable intervention programs that can better address the obesogenic environment and critical periods in the life course to tackle childhood overweight and obesity.

## Data Availability

All data underlying the results are available as part of the article and no additional source data are required. Zenodo: PRISMA-ScR checklist for “Causes and contexts of childhood overweight and obesity in the Pacific region: a scoping review”. https://doi.org/10.5281/zenodo.7582781 ^50^. Zenodo: Flowchart for “Causes and contexts of childhood overweight and obesity in the Pacific region: a scoping review”. https://doi.org/10.5281/zenodo.7566959 ^49^. Data are available under the terms of the Creative Commons Zero "No rights reserved" data waiver (CC0 1.0 Public domain dedication). **Ethics and consent:** Ethical approval and consent were not required.

## Footnotes

^1^ Republic of Marshall Islands, Federated States of Micronesia and Palau are US associated states. Niue and Cook Islands are New Zealand associated states. New Caledonia, Wallis-et-Futuna and French Polynesia are French overseas territories, Pitcairn is a British territory and American Samoa and Guam are US territories.
