# Supplementary material for: Prevalence, causes and contexts of childhood overweight and obesity in the Pacific region: a scoping review
Source: Open Res Eur. 2023 Nov 20;3:52. Originally published 2023 Apr 3. [Version 2] doi: 10.12688/openreseurope.15361.2 (PMC10685071; doi:10.12688/openreseurope.15361.2)
Supplement: Peer review history

# Reviewer report: Reviewer response for version 2

**DOI:** 10.21956/openreseurope.18134.r36314

**Published:** 2023-11-28

**Authors:** Haley L. Cash

**Affiliations:** 1 Pacific Islands Health Officers Association, Honolulu, HI, USA

The original comments have been properly addressed.

Are the rationale for, and objectives of, the Systematic Review clearly stated?

Partly

Is the statistical analysis and its interpretation appropriate?

Not applicable

Are sufficient details of the methods and analysis provided to allow replication by others?

Yes

Are the conclusions drawn adequately supported by the results presented in the review?

Yes

Reviewer Expertise:

non-communicable disease epidemiology

I confirm that I have read this submission and believe that I have an appropriate level of expertise to confirm that it is of an acceptable scientific standard.

# Reviewer report: Reviewer response for version 2

**DOI:** 10.21956/openreseurope.18134.r36313

**Published:** 2023-11-24

**Authors:** Marie Pierre Moisan, Rachel Ginieis

**Affiliations:** 1 Nutrition et Neurobiologie Intégrée, UMR 1286, University of Bordeaux, Bordeaux, France; 2 Nutrition and Neurobiologie Intégrée (Nutrineuro), UMR 1286, NutriPsy team, University of Bordeaux, Talence, Nouvelle-Aquitaine, France

The authors have answered satisfactorily to the requests. We do not have further comments.

Are the rationale for, and objectives of, the Systematic Review clearly stated?

Yes

Is the statistical analysis and its interpretation appropriate?

Not applicable

Are sufficient details of the methods and analysis provided to allow replication by others?

Yes

Are the conclusions drawn adequately supported by the results presented in the review?

Yes

Reviewer Expertise:

Nutrition, obesity, hormones

We confirm that we have read this submission and believe that we have an appropriate level of expertise to confirm that it is of an acceptable scientific standard.

# Reviewer report: Reviewer response for version 1

**DOI:** 10.21956/openreseurope.16606.r33245

**Published:** 2023-07-21

**Authors:** Marie Pierre Moisan, Rachel Ginieis

**Affiliations:** 1 Nutrition et Neurobiologie Intégrée, UMR 1286, University of Bordeaux, Bordeaux, France; 2 Nutrition and Neurobiologie Intégrée (Nutrineuro), UMR 1286, NutriPsy team, University of Bordeaux, Talence, Nouvelle-Aquitaine, France

The manuscript of Bertand-Protat et al is a scoping review aiming at presenting a comprehensive review of all available information regarding overweight and obesity prevalence in the Pacific islands countries and territories for children aged between 6 and 12, as well as causes and contexts. The methodology used for the review is well exposed and the data clearly presented. Although the number of selected articles is rather low, this report is very interesting. Indeed, this population of children in this part of the world is understudied although the prevalence of overweight and obesity is alarmingly high, reaching over 55% in some of the Pacific islands countries and territories. In addition to the prevalence, the authors have complied and then discussed the determinants of overweight and obesity in the different Pacific islands studied, providing an interesting basis for future investigations and interventional studies. The limitations of the various studies, and therefore of the review, have also been discussed by the authors.

 Here are some comments aiming to improve the manuscript:

- The title starts with “causes and contexts of childhood OWOB…” while a great part of the review is on the prevalence of OWOB. It seems more appropriate to start the title with “Prevalence, causes and contexts of childhood..”
- In the plain language summary, the authors write “This scoping review aims to understand the causes of overweight and obesity prevalence for children aged six to 12 years old living in the Pacific region and identified possible related causes.” This sentence needs to be re-phrased. I would suggest again here to present the work on prevalence and then causes and contexts.
- A definition is given for overweight and obesity ( *“Overweight and obesity are defined as abnormal or excessive fat accumulation that may impair health.”) *but the 2 conditions are not distinguished although they have different health consequences. This should be clarified and references of the definition cited.
- The review includes articles from 1980 but the rationale of this criteria is not explained. Does it has to do with the “faster access to modern life in the past few decades” mentioned in the plain language summary?
- in the introduction, end of second paragraph, it would be interesting to have the figures of mean BMI in children in the Pacific region.
- Similarly, at the beginning of the 4 paragraph of introduction , inclusion of figures for BMI would provide more concrete information.
- In the method, it is not mention clearly if PRISMA for Scoping Reviews methodology was used.
- In figure 2 Google scholar use is not mentioned although it is mentioned in the text; please explain if the data were redundant with Pubmed or else?
- In the results section, the tool for assessing BMI is not mentioned so it is difficult to know how comparisons have been made (eg if the data come from different tools such as CDC or French BMI references standards).
- Figures would be appreciated rather than comparisons such as “ Native Hawaiian boys aged 8–9 years old were found to be significantly more overweight than their classmates ^”^
- Please indicate age of children in grade 2 or 3 or 5 in the sentence “In American Samoa, the OWOB rate was 41.3% for grade two children, 43.9% for grade three and 50% for grade five ^29^. It was also found that sex might influence weight status.”
- In the discussion, replace “systematic review” by “scoping review”
- More emphasis could be done on the fact that in some countries BMI is positively correlated with higher parents’ income (USAPI) or education (Guam) and attending private school (Marshall islands) while in other islands the correlation is negative as often reported in most countries of the world.
- More emphasis could be made also on the limitations of BMI measure. For example it is known that muscular person will have a high BMI, therefore overestimating their fat mass. This can be corrected with HDL cholesterol and waist circumference measures. Similarly person with low stature would have a low BMI when their fat mass might be high.

Are the rationale for, and objectives of, the Systematic Review clearly stated?

Yes

Is the statistical analysis and its interpretation appropriate?

Not applicable

Are sufficient details of the methods and analysis provided to allow replication by others?

Yes

Are the conclusions drawn adequately supported by the results presented in the review?

Yes

Reviewer Expertise:

Nutrition, obesity, hormones

We confirm that we have read this submission and believe that we have an appropriate level of expertise to confirm that it is of an acceptable scientific standard, however we have significant reservations, as outlined above.

# Response

**Published:** 2023-10-27

**Authors:** olivier Galy

**Affiliations:** university of new caledonia, New Caledonia

**Authors: **We thank the reviewer for it appraisals. We have done our best to address the concerns and hope that the manuscript is now improved.

**1. The title starts with “causes and contexts of childhood OWOB…” while a great part of the review is on the prevalence of OWOB. It seems more appropriate to start the title with “Prevalence, causes and contexts of childhood..”**

 Authors : title modified  

**2. In the plain language summary, the authors write “This scoping review aims to understand the causes of overweight and obesity prevalence for children aged six to 12 years old living in the Pacific region and identified possible related causes.” This sentence needs to be re-phrased. I would suggest again here to present the work on prevalence and then causes and contexts.**

 Authors: plain summary has been rephrased as suggested. Please see Page 4 lines 7 to 9    

**3. A definition is given for overweight and obesity (“Overweight and obesity are defined as abnormal or excessive fat accumulation that may impair health.”) but the 2 conditions are not distinguished although they have different health consequences. This should be clarified and references of the definition cited.**

 Authors: this is WHO definition. This information has been added to the manuscript. Please see Page 5 line 24        

**4. The review includes articles from 1980 but the rationale of this criteria is not explained. Does it has to do with the “faster access to modern life in the past few decades” mentioned in the plain language summary?**

 Authors: The rationale is the availability of articles related to the topic of children OWOB in the Pacific region on databases.    

**5. in the introduction, end of second paragraph, it would be interesting to have the figures of mean BMI in children in the Pacific region**

 Authors: we can’t calculate children mean BMI for the whole region because the BMI references used are different (WHO, IOTF, CDC, French references).    

**6. Similarly, at the beginning of the 4 paragraph of introduction, inclusion of figures for BMI would provide more concrete information**

 Authors: same comment as above. Data are not comparable so we can’t provide figures.    

**7. In the method, it is not mentioned clearly if PRISMA for Scoping Reviews methodology was used.**

 Authors: yes, Preferred Reporting Items for Systematic reviews and Meta-Analyses extension for Scoping Reviews (PRISMA-ScR) Checklist was used. This information is provided on page 26, lines129-130.    

**8. In figure 2 Google scholar use is not mentioned although it is mentioned in the text; please explain if the data were redundant with Pubmed or else?**

 Authors: No relevant articles were found in google scholar, apart from those available on Pubmed.  

**9. In the results section, the tool for assessing BMI is not mentioned so it is difficult to know how comparisons have been made (eg if the data come from different tools such as CDC or French BMI references standards).**

 Authors: It is not possible to make clear comparison between countries or subregions due to this lake of standardization in tools, methodology, age group, etc. as explained on lines 160-170. However, we’ve decided to present the most relevant data.  

**10. Figures would be appreciated rather than comparisons such as “ Native Hawaiian boys aged 8–9 years old were found to be significantly more overweight than their classmates** **^”^**

 Authors: Thanks for this suggestion but after discussion with co-authors we’ve decided that there is enough tables and figures in the article.    

**11. Please indicate age of children in grade 2 or 3 or 5 in the sentence “In American Samoa, the OWOB rate was 41.3% for grade two children, 43.9% for grade three and 50% for grade five. It was also found that sex might influence weight status.”**

 Authors: This information is not provided in the article where data has been extracted.    

**12. In the discussion, replace “systematic review” by “scoping review”.**

 Authors: Done Please see Page 29, line 259.    

**13. More emphasis could be done on the fact that in some countries BMI is positively correlated with higher parents’ income (USAPI) or education (Guam) and attending private school (Marshall islands) while in other islands the correlation is negative as often reported in most countries of the world.**

 Authors: Due to different methodologies used we’ve decided to not provide more details/explanation on this point.    

**14. More emphasis could be made also on the limitations of BMI measure. For example it is known that muscular person will have a high BMI, therefore overestimating their fat mass. This can be corrected with HDL cholesterol and waist circumference measures. Similarly person with low stature would have a low BMI when their fat mass might be high.**

 Authors: we’ve chosen to follow WHO guidelines and recommendations to evaluate children’s BMI.

# Reviewer report: Reviewer response for version 1

**DOI:** 10.21956/openreseurope.16606.r31129

**Published:** 2023-05-25

**Authors:** Haley L. Cash

**Affiliations:** 1 Pacific Islands Health Officers Association, Honolulu, HI, USA

This article provides a valuable review of childhood overweight/obesity in the Pacific region. Overall, this review is thorough and well written, though it could benefit from some minor revisions. Please see specific comments below.

**General:**

 The objective of this review needs to be clearly stated and used consistently throughout the article. Also, the author could consider expanding to two aims: 1) summarizing the prevalence of overweight/obesity among 6–12-year-olds in the Pacific and 2) summarizing the known causes of overweight/obesity among 6-12-year-olds in the Pacific. 

**Introduction:**

 The introduction contains a lot of interesting and valuable information; however, this section could be better organized. More justification is needed to explain this target age group. Also, a more robust epidemiological background on overweight/obesity in the Pacific would be useful. There are some data from Pacific Islanders living in New Zealand, but no data from the Pacific islands. It is recommended that overweight/obesity data from adults and adolescents be provided to provide context for this issue in the region.

 This section also states that collecting anthropometric data from Pacific youth is a priority, although there are limited data available, therefore it makes sense that summarizing available data is also an objective of this review.

**Results:**

- In the first paragraph on page 12, please revise the verbiage to state “northern jurisdictions”, and “US territories, commonwealths, and freely associate states”.
- In paragraph 3 on page 12, please specify age groups for all OWOB prevalence data listed, as these vary slightly in each jurisdiction.
- Figure 3 is not necessary, please consider removing.
- Figure 4 is quite small but provides a useful summary, consider making this larger.
- The results section summarizes OWOB in the Pacific, so this should be a second objective of this review.

**Discussion:**

- This section begins with a summary of OWOB prevalence data based on this review, so again, this should be considered as an objective.
- In paragraph 1 of this section, it is stated that “ *high prevalence of OWOB in the region was mainly explained by…*”. Please consider rewording considering that there is no research that can fully understand this complex issue. Perhaps state that “most commonly observed factors associated with childhood OWOB are…”.
- Another limitation to lack of childhood OWOB data in the Pacific is the actual reporting of collected data. Many jurisdictions are collecting these data, but they are not being analyzed and reported, especially in publicly available reports. Consider adding this as a limitation.

Are the rationale for, and objectives of, the Systematic Review clearly stated?

Partly

Is the statistical analysis and its interpretation appropriate?

Not applicable

Are sufficient details of the methods and analysis provided to allow replication by others?

Yes

Are the conclusions drawn adequately supported by the results presented in the review?

Yes

Reviewer Expertise:

non-communicable disease epidemiology

I confirm that I have read this submission and believe that I have an appropriate level of expertise to confirm that it is of an acceptable scientific standard, however I have significant reservations, as outlined above.

# Response

**Published:** 2023-10-27

**Authors:** olivier Galy

**Affiliations:** university of new caledonia, New Caledonia

**Authors: **We thank the reviewer for it appraisals. We have done our best to address the concerns and hope that the manuscript is now improved.  

**1.** **The objective of this review needs to be clearly stated and used consistently throughout the article. Also, the author could consider expanding to two aims: 1) summarizing the prevalence of overweight/obesity among 6–12-year-olds in the Pacific and 2) summarizing the known causes of overweight/obesity among 6-12-year-olds in the Pacific.** 

 Authors: We have rephrased some sentences in response to this recommendation. Please see Page 6 (last paragraph of the introduction)    

**2.** **A more robust epidemiological background on overweight/obesity in the Pacific would be useful. There are some data from Pacific Islanders living in New Zealand, but no data from the Pacific islands. It is recommended that overweight/obesity data from adults and adolescents be provided to provide context for this issue in the region.**

 Authors: We have added 2 sentences in response to this recommendation. Please see Page 5 line 44    

**3.** **In the first paragraph on page 12, please revise the verbiage to state “northern jurisdictions”, and “US territories, commonwealths, and freely associate states”.**

 Authors: done. Please see Page 27 lines 166 to 168      

**4. In paragraph 3 on page 12, please specify age groups for all OWOB prevalence data listed, as these vary slightly in each jurisdiction.**

 Authors: done. Please see Page 27 lines 177 to 179    

**5. Figure 3 is not necessary, please consider removing.**

 Authors: done. Figure has been removed.    

**6. Figure 4 is quite small but provides a useful summary, consider making this larger.**

 Authors: this is a comment that can be addressed by the editorial office only.    

**7. The results section summarizes OWOB in the Pacific, so this should be a second objective of this review.**

 Authors : This was addressed thru comment 1.    

**8. This section begins with a summary of OWOB prevalence data based on this review, so again, this should be considered as an objective.**

 Authors : This was addressed thru comment 1.    

**9. In paragraph 1 of this section, it is stated that “high prevalence of OWOB in the region was mainly explained by…”. Please consider rewording considering that there is no research that can fully understand this complex issue. Perhaps state that “most commonly observed factors associated with childhood OWOB are…”.**

 Authors: sentence modified. Please see Page 29 line 263-265          

**10. Another limitation to lack of childhood OWOB data in the Pacific is the actual reporting of collected data. Many jurisdictions are collecting these data, but they are not being analyzed and reported, especially in publicly available reports. Consider adding this as a limitation.**

 Authors: sentence modified. Please see Page 32 line 399-401
